# Ten problems in Gödel logic

**DOI:** 10.1007/s00500-016-2366-9

**Published:** 2016-09-28

**Authors:** Juan P. Aguilera, Matthias Baaz

**Affiliations:** Vienna University of Technology, Wiedner Hauptstraße 8-10, 1040 Vienna, Austria

**Keywords:** Classical Logic, Intuitionistic Logic, Predicate Symbol, Existential Quantifier, Kripke Frame

## Introduction

Gödel logics are an important class of intermediate logics with connections to many areas and applications of logic such as temporal logic, Heyting algebras, fuzzy logic, and parallel processing. In this paper, we present ten open problems in the proof and model theories of Gödel Logic. The problems can be seen to be ordered both thematically and by generality. Some of the problems have been open for more than thirty years. The second author discussed many of them with Franco Montagna, to whose memory this paper is dedicated.

## Preliminaries

Gödel logics are extensions of intuitionistic logic that take truth values in a closed subset of the interval [0, 1]. We denote by $G_V$ the Gödel logic whose truth-value set is *V*.

### Definition 1

A *valuation* ${\mathfrak I}$ for the Gödel logic $G_V$ is

1. a nonempty set $U = U^{\mathfrak I}$, the ‘universe’ of ${\mathfrak I}$,
2. for each *k*-ary predicate symbol *P*, a function $P^{\mathfrak I}:U^k \rightarrow V$,
3. for each *k*-ary function symbol *f*, a function $f^{\mathfrak I}:U^k \!\rightarrow \! U$,
4. for each variable *v*, a value $v^{\mathfrak I}\in U$.

Any interpretation ${\mathfrak I}$ can be naturally extended to define a value $t^{\mathfrak I}$ for any term *t* and a truth value ${\mathfrak I}(A)$ for any formula *A* of the language $\mathcal {L}^U$ (obtained by extending the base language with names for each element in *U*). For a term $t = f(u_1, \ldots , u_k)$ we define ${\mathfrak I}(t)=f^{\mathfrak I}(u_1^{\mathfrak I}, \ldots , u_k^{\mathfrak I})$. For atomic formulae we define ${\mathfrak I}(\bot ) = 0$ and ${\mathfrak I}(P(t_1,\dots , t_n)) = P^{\mathfrak I}(t_1^{\mathfrak I}, \ldots , t_n^{\mathfrak I})$. For composite formulae *A* we set inductively:

$$\begin{aligned} {\mathfrak I}(A\wedge B)&= \min \{{\mathfrak I}(A),{\mathfrak I}(B)\},\\ {\mathfrak I}(A\vee B)&= \max \{{\mathfrak I}(A),{\mathfrak I}(B)\},\\ {\mathfrak I}(A\rightarrow B)&= {\left\{ \begin{array}{ll}{\mathfrak I}(B) &{} \text {if} \,\,{\mathfrak I}(A) > {\mathfrak I}(B),\\ 1 &{} \text {if}\,\, {\mathfrak I}(A) \le {\mathfrak I}(B).\end{array}\right. } \\ {\mathfrak I}(\forall x\, A(x))&= \inf \{{\mathfrak I}(A(u)) :u \in U\}\\ {\mathfrak I}(\exists x\, A(x))&= \sup \{{\mathfrak I}(A(u)) :u \in U\}. \end{aligned}$$

We call a formula *valid* with respect to *V* if it is mapped to 1 for all valuations based on *V*. A formula is *satisfiable* with respect to *V* if it is mapped to 1 for some valuation based on *V*. The set of all valid formulae with respect to *V* is often identified with ${ G }_V$.

$\lnot A$ is often defined by $A \rightarrow \bot$. Thus, we have

$$\begin{aligned} {\mathfrak I}(\lnot A)&= {\left\{ \begin{array}{ll} 0 &{} \text {if}\,\, {\mathfrak I}(A)>0,\\ 1 &{} \text {otherwise,}\end{array}\right. } \end{aligned}$$

The following formulae play an important role when axiomatizing Gödel logics:

$$\begin{aligned} \textsc {qs}&\quad \forall x(C\vee A(x)) \rightarrow (C\vee \forall xA(x)) \\ \textsc {lin}&\quad (A\rightarrow B)\vee (B\rightarrow A)\\ \textsc {iso}_0&\quad \forall x\lnot \lnot A(x) \rightarrow \lnot \lnot \forall xA(x) \end{aligned}$$

where *x* is not free in *C*. Their names can be explained as follows: $\textsc {qs}$ stands for ‘quantifier shift,’ $\textsc {lin}$ for ‘linearity,’ $\textsc {iso}_0$ for ‘isolation axiom of 0.’

A few prototypical Gödel logics are worth defining. ${ G_{\textsc {iso}_0} }$ denotes the logic with truth-value set $\{0\} \cup [\frac{1}{2}, 1]$, ${ G_\uparrow }$ denotes the logic with truth-value set $\{1 - \frac{1}{n + 1}: n \in \mathbb {N}\} \cup \{1\}$, and ${ G_\downarrow }$ denotes the logic with truth-value set $\{0\} \cup \{\frac{1}{n + 1}: n \in \mathbb {N}\}$. ${ G_{[0, 1]} }$ is axiomatized by adding axioms $\textsc {qs}$ and $\textsc {lin}$ to any axiomatization of intuitionistic logic; ${ G_{\textsc {iso}_0} }$ is axiomatized by adding $\textsc {iso}_0$ to an axiomatization of ${ G_{[0, 1]} }$ (see Baaz et al. 2008).

A Gödel logic $G_V$ can be extended by a *projection* operator $\varDelta$, to be interpreted as

$$\begin{aligned} {\mathfrak I}({\mathop {\triangle }}A)&= {\left\{ \begin{array}{ll} 1 &{} {\mathfrak I}(A) = 1,\\ 0 &{} {\mathfrak I}(A) < 1.\end{array}\right. } \end{aligned}$$

Whenever we do so, we denote the resulting logic by $G_V^\varDelta$.

## Problems

The first problems we discuss relate to the proof theory of Gödel logics.

### Problem 1

Provide an analytic calculus for ${ G_{\textsc {iso}_0} }$.

In this context, we might identify analyticity with exhibiting an adequate subformula property. Recall that ${ G_{\textsc {iso}_0} }$ is recursively enumerable (see above).

A possible way to solve this problem might be to first provide an analytic calculus for intuitionistic logic extended by the schema $\textsc {iso}_0$ and then embedding it into an analytic hypersequent calculus deriving $\textsc {lin}$ and $\textsc {qs}$ (see Baaz et al. 1998). Such an extension of intuitionistic logic could be based on weakened eigenvariable conditions that are sound for classical logic by considering that every negated subformula of a formula is classical in presence of $\textsc {iso}_0$.

This approach seems promising, as a hypersequent calculus based on intuitionistic logic has been shown to be an adequate analytic calculus for $G_{[0,1]}$ (see Baaz et al. 2003). Indeed, the logic $G_{[0,1]}$ is clearly better understood. Nonetheless, several questions about it are yet to be answered. An example of this is the issue of interpolation.

### Problem 2

Determine whether $G_{[0,1]}$ admits interpolation.

Propositional Gödel logic is one of the seven intermediate logics that admit interpolation (Maksimova 1979). Moreover, it admits strong interpolation, as propositional quantifiers are eliminable in propositional quantifier logic. By a result of Mints et al. (2013), intuitionistic logic augmented with $\textsc {qs}$ does not interpolate. In contrast to this, other extensive fragments of ${ G_{[0, 1]} }$ do admit interpolation: let $G^-_{[0,1]}$ be the fragment of $G_{[0,1]}$ consisting of all formulae of the form

1 $$\begin{aligned} \bigwedge _i A_i \rightarrow \bigvee _i B_i, \end{aligned}$$

such that each $A_i$ and $B_i$ are prenex except perhaps for universal quantifiers on the left-hand side or existential quantifiers on the right-hand side. We call those quantifiers *weak quantifiers*. If $\varphi$ is of the form (1), we call its antecedent the *negative* part of $\varphi$ and its consequent the *positive* part.

### Proposition 2

$$G^-_{[0,1]}$$ admits interpolation

### Proof

(Proof sketch.) Let $\varphi$ be a formula of the form (1) valid (and therefore derivable) in $G_{[0,1]}$. First, we replace strong quantifiers by adequate Skolem functions. We calculate the Herbrand expansion of $\varphi$ using cut-elimination of the hypersequent calculus for $G_{[0,1]}$ (in the Herbrand expansion, every universal quantifier on the negative part is replaced by a conjunction and every existential quantifier on the positive part is replaced by a disjunction). Calculate an interpolant for this valid propositional formula. Finally, remove the Skolem terms from the negative part (resp. positive) and the interpolant simultaneously in a way similar to the Second Epsilon Theorem (see Hilbert and Bernays 1970).

A related problem is the following:

### Problem 3

Develop a suitable Skolemization for ${ G_{[0, 1]} }$.

The prenex fragment of ${ G_{[0, 1]} }$ admits Skolemization (Baaz et al. 2001). Intuitionistic logic with the addition of an existence predicate also admits Skolemization (Baaz and Iemhoff 2006). This Skolemization cannot be extended directly to Gödel logics because the additional axioms $\textsc {lin}$ and $\textsc {qs}$ used in a proof are in general not reducible to finitely many universal formulae without extending the base language.

### Problem 4

Construct a conservative epsilon calculus representing ${ G_{[0, 1]} }$.

The problem arises from the fact that the usual critical formulae of classical logic $A(t) \rightarrow A(\varepsilon _x A(x))$ (see Hilbert and Bernays 1970) are not conservative, i.e., it is not the case that only the translations of valid formulae are derivable. For instance:

### Proposition 3

The critical formulae imply the validity of $\exists x (P(f(x)) \rightarrow P(x))$.

### Proof

The two critical formulae

$$\begin{aligned} P(f(\varepsilon _x P(x))) \rightarrow P(\epsilon _x P(x))&\\ \left( P(f(\varepsilon _x P(x))) \rightarrow P(\epsilon _x P(x)) \right)&\rightarrow \left( P\left( f(e ) \right) \rightarrow P(e) \right) , \end{aligned}$$

where

$$\begin{aligned} e = \varepsilon _x(P(f(x)) \rightarrow P(x)). \end{aligned}$$

by modus ponens imply $P\left( f(e ) \right) \rightarrow P(e)$, the translation of $\exists x (P(f(x)) \rightarrow P(x))$, which is not valid in ${ G_{[0, 1]} }$.

Our next problem deals with an extension of Gödel logic. Specifically, consider a notion of identity extending interpretations $\mathfrak {I}$ such that

$$\begin{aligned} \mathfrak {I}(s=t) = 1 \text { if, and only if, } \mathfrak {I}(s) = \mathfrak {I}(t). \end{aligned}$$

### Proposition 4

The upward Löwenheim–Skolem theorem fails for $G^\varDelta _V$ whenever *V* is infinite.

### Proof

Consider the formula

2 $$\begin{aligned} \forall x\forall y \left( \lnot \varDelta (x = y) \rightarrow \lnot \varDelta \left( P(x) \leftrightarrow P(y) \right) \right) . \end{aligned}$$

If *V* is of infinite cardinality $\kappa$, then the formula has a model of cardinality $\kappa$, but no model of higher cardinality.

According to the above proof, $2^{\aleph _0}$ is an upper bound on the cardinalities of models of (2). The following result is well known (see, for example, Bell and Slomson 1969).

### Proposition 5

(Hanf) For any language $\mathcal {L}$ whose sentences form a set *S*, there exists a cardinal $\kappa$—the Hanf number of $\mathcal {L}$—such that for all $\varphi \in S$, if $\varphi$ has a model of cardinality $\lambda \ge \kappa$, then $\varphi$ has a model of arbitrarily large cardinalities.

### Proof

For each $\varphi$ such that the cardinalities of models of $\varphi$ are bounded, let $\kappa _\varphi$ be the least upper bound. Then $\kappa$ is the supremum of the set of all $\kappa _\varphi$.

### Problem 5

Determine the Hanf number of $G^\varDelta _V$, for *V* infinite.

We remark that without the operator $\varDelta$, the Löwenheim–Skolem theorem holds, because the Gödel logics correspond to finite or countable linear Kripke frames with constant domains (Beckmann and Preining 2007).

Problem 6 and Problem 8 below concern recursive enumerability of various Gödel logics.

### Definition 6

Let $G_V$ be a propositional or first-order Gödel logic. We define $G^P_V$ to be the extension of $G_V$ by propositional quantifiers $\dot{\forall }$, $\dot{\exists }$. Specifically, for any valuation $\mathfrak {I}$, we set $\mathfrak {I}(\dot{\forall }X A(X))$ (resp. $\mathfrak {I}(\dot{\exists }X A(X))$) to be the infimum (resp. supremum) of $\mathfrak {J}(A(X))$ taken over all valuations $\mathfrak {J}$ that coincide with $\mathfrak {I}$, except maybe on $\mathfrak {I}(X)$. We use capital letters to denote propositional variables.

### Problem 6

Is first-order ${ G^P_{[0, 1]} }$ recursively enumerable?

We note that first-order ${ G^P_{[0, 1]} }$ admits conversion to prenex normal forms.

### Proposition 7

Each formula in ${ G^P_{[0, 1]} }$ is equivalent to a formula in prenex normal form.

### Proof

It is easy to check that the following equivalences are valid:

$$\begin{aligned} (\forall x A(x) \rightarrow B)&\leftrightarrow \dot{\forall }Z \exists x (A(x) \rightarrow Z \vee Z \rightarrow B) \\ (\dot{\forall } X A(X) \rightarrow B)&\leftrightarrow \dot{\forall }Z \dot{\exists } X (A(X) \rightarrow Z \vee Z \rightarrow B)\\ (A \rightarrow \exists x B(x))&\leftrightarrow \dot{\forall }Z \exists x(A \rightarrow Z \vee Z \rightarrow B(x))\\ (A \rightarrow \dot{\exists } X B(X))&\leftrightarrow \dot{\forall }Z \dot{\exists } X(A \rightarrow Z \vee Z \rightarrow B(X)) \end{aligned}$$

As all other classically valid quantifier shifts are valid in all Gödel logics, the result follows.

A consequence of a positive solution to Problem 6 would be that if one could show that first-order ${ G^P_{[0, 1]} }$ admits Herbrand disjunctions, then one could construct suitable Skolem functionals for ${ G^P_{[0, 1]} }$.

Propositional quantifiers are eliminable in propositional ${ G^P_{[0, 1]} }$ (Baaz and Veith 1998). This logic is the only quantified propositional Gödel logic with this property. However, propositional ${ G_\uparrow }$ (resp. ${ G_\downarrow }$) admit propositional quantifier elimination if an additional one-place connective is added (Baaz et al. 2000; Baaz and Preining 2008), namely $\circ Y = \dot{\forall }X \big ((X \rightarrow Y) \vee X\big )$ (resp. $\dot{\forall }X\big ((X \rightarrow Y)\rightarrow X\big )$). This connective assigns to *Y* the immediate successor (resp. predecessor) of its value.

### Problem 7

Characterize the propositional Gödel logics that admit quantifier elimination if an additional one-place connective is added.

We can also restrict our attention to monadic fragments of Gödel logic.

### Problem 8

Characterize the Gödel logics whose monadic fragments are recursively enumerable.

Clearly, the monadic fragments of ${ G_{[0, 1]} }$ and ${ G_{\textsc {iso}_0} }$ are r.e. On the other hand, the monadic fragment of ${ G_\uparrow }$ is not r.e. as any nonvalid sentence has a finitely valued countermodel and the monadic fragment of ${ G_\uparrow }$ is undecidable (see Baaz and Preining 2016). In particular, we might wonder:

### Problem 9

Is there a Gödel logic that is undecidable when restricted to its fragment with a single one-place predicate symbol?

It is known that if we restrict ourselves to monadic fragments with one predicate symbol, there are countably many Gödel logics of increasing complexity (see Beckmann and Preining 2015). These fragments can be considered as quantified propositional logics where the actual set of truth values is determined by the interpretation.

Up to now, we have only discussed problems of validity. The representation of Gödel logics with respect to validity can be always reduced to the sets of valid sentences. However, if we speak of validity and satisfiability in parallel, this representation is useless and we therefore have to refer to the truth-value sets. This is unfortunate, as uncountably many different Gödel logics with respect to truth-value sets correspond to countably many sets of valid sentences (Beckmann et al. 2008). The following problem is a particular instance of this phenomenon.

### Problem 10

There are Gödel logics which differ according to the sets of their valid sentences whose sets of satisfiable sentences coincide. For example, ${ G_{\textsc {iso}_0} }$ has the same satisfiable sentences as classical logic. Does the converse hold?
